# Factors affecting bone maturation in Chinese girls aged 4–8 years with isolated premature thelarche

**DOI:** 10.1186/s12887-020-02256-w

**Published:** 2020-07-29

**Authors:** Huiping Su, Zhe Su, Lili Pan, Li Wang, Zhongwei Xu, Gang Peng, Xianglei Li

**Affiliations:** 1 Department of Endocrinology, Shenzhen Children’s Hospital, No. 7019, Yitian Road, Futian District, 518038 Shenzhen, Guangdong Province People’s Republic of China; 2 Department of Adolescent Gynecology, Shenzhen Children’s Hospital, Shenzhen, China

**Keywords:** Premature thelarche, Bone maturation, Insulin-like growth factor-1, Dehydroepiandrosterone sulfate, Body mass index

## Abstract

**Background:**

In isolated premature thelarche (IPT) girls, bone age (BA) is considered consistent with chronological age. However, some IPT girls confirmed by gonadotropin-releasing hormone (GnRH) stimulation test could show another trend. We analysed BA and possible potentiating factors in a selected group of girls aged 4–8 years with IPT.

**Methods:**

IPT girls confirmed by GnRH stimulation test aged 4–8 years hospitalized from January 2015 to April 2018 at Shenzhen Children’s Hospital were included in this retrospective study. They were divided into two groups with advanced BA of 2 years as the cut-off. Body mass index (BMI) and hormone levels were the main outcome measures, and regression analysis was used to identify independent risk factors. IPT girls were divided into subgroups according to the levels of BMI standard deviation score (SDS), insulin-like growth factor-1 (IGF-1) SDS and dehydroepiandrosterone sulfate (DHEAS) SDS for comparisons of advanced BA.

**Results:**

Overall, 423 subjects were included and classified into the advanced BA group (48.7%, *n* = 206) and control group (51.3%, *n* = 217). The advanced BA group had significantly higher BMI SDS, serum DHEAS SDS, IGF-1 SDS, androstenedione and fasting insulin and significantly lower sex hormone binding globulin (all *p* < 0.001). Serum IGF-1 SDS (OR = 1.926, *p*<0.001), BMI SDS (OR = 1.427, *p* = 0.001) and DHEAS SDS (OR = 1.131, *p* = 0.005) were independent risk factors for significantly advanced BA. In the multiple linear regression model, serum IGF-1 SDS, BMI SDS and DHEAS SDS were the strongest predictors of advanced BA, accounting for 19.3% of the variance. According to BMI, 423 patients were classified into three groups: normal weight (56.03%, *n* = 237), overweight (19.15%, *n* = 81) and obesity (24.82%, *n* = 105). The proportion of advanced BA in obesity group was significantly higher than those of normal weight and overweight groups (*χ*^2^ = 18.088, *p*<0.001). In the subgroup with normal weight, higher serum IGF-1 SDS (*p* = 0.009) and DHEAS SDS (*p* = 0.003) affected BA advancement independent of BMI SDS.

**Conclusions:**

Girls with IPT confirmed by GnRH stimulation test aged 4–8 years might have significantly advanced BA. Obesity was highly associated with advanced BA. Age-specific serum IGF-1 SDS and DHEAS SDS were risk factors for BA advancement independent of BMI.

## Background

Isolated premature thelarche (IPT) is defined as isolated breast development without the development of other sexual characteristics in girls before 8 years of age. IPT usually presents before the age of 2, and approximately 83.1% of patients show spontaneous regression in 2 years, which may be attributable to the so-called mini-puberty [1]. IPT presented in an elder age is also considered to be a benign and self-limiting phenomenon that usually not influence the growth or timing of puberty. Generally, bone age (BA) in IPT girls is consistent with chronological age (CA) [2–4].

However, Stanhope and Brook [5] first described a new clinical situation of IPT in 1990. It was characterized by BA advancement and/or growth acceleration and hypothesized that different hormonal changes probably occur. In 1998, Volta et al. [6] reported that 34 (28.6%) of 119 girls aged 1–8 years with IPT presented with similar characteristics. So, IPT is not always benign and some may have advanced BA. However, the previous studies had small sample size and infant cases were included, without the further analysis of possible factors affecting BA in IPT.

In clinical practice, we also found that some IPT girls aged 4–8 years who need for further investigations had BA advancement of more than 2 years. Oestradiol levels, the most important factor affecting BA, are within the normal prepubertal range in IPT girls [7]. The reason for advanced BA in this group remains unclear.

Therefore, the present study aimed to analyse the characteristics of bone maturation in Chinese girls aged 4–8 years with IPT and the possible potentiating factors for advanced BA in IPT.

##  Methods

### Study design and participant selection

Most of girls aged 4–8 years with breast development were followed up in our outpatient clinic. They were potentially hospitalized for gonadotropin-releasing hormone (GnRH) stimulation test when they presented with no breast tissue regression for at least three months and one or more of the following: (1) Progressive breast development: the progression of breast Tanner stage from one to another was less than half a year; (2) Significant BA advancement: BA was greater than 2 years above CA; (3) Linear growth acceleration: height velocity was above the expected value for gender and age and/or above the familial genetic channel. We identified IPT girls who were confirmed by GnRH stimulation test in the Endocrinology Department of Shenzhen Children’s Hospital from January 2015 to April 2018.

This was a retrospective case-control study. The cases consisted of IPT girls with significantly advanced BA (BA minus CA (BA-CA) ≥ 2 years). The controls were IPT girls without significantly advanced BA (BA-CA < 2 years).

The inclusion criteria were as follows: (1) Age at onset of the disease was 4–8 years; (2) Patients with isolated breast development; (3) Patients without other secondary sexual characteristics, pigmentation of the nipple and areola, and growth of pubic hair or armpit hair; (4) Peak luteinizing hormone (LH) level was less than 5 IU/L and the ratio of peak LH to peak follicle-stimulating hormone (FSH) in the GnRH stimulation test was less than 0.6; (5) Breast development had no other causes, such as peripheral precocious puberty, exogenous estrogen intake, local mammary gland hyperplasia and so on. There had been 480 IPT girls included in the study initially.

**Fig. 1:**
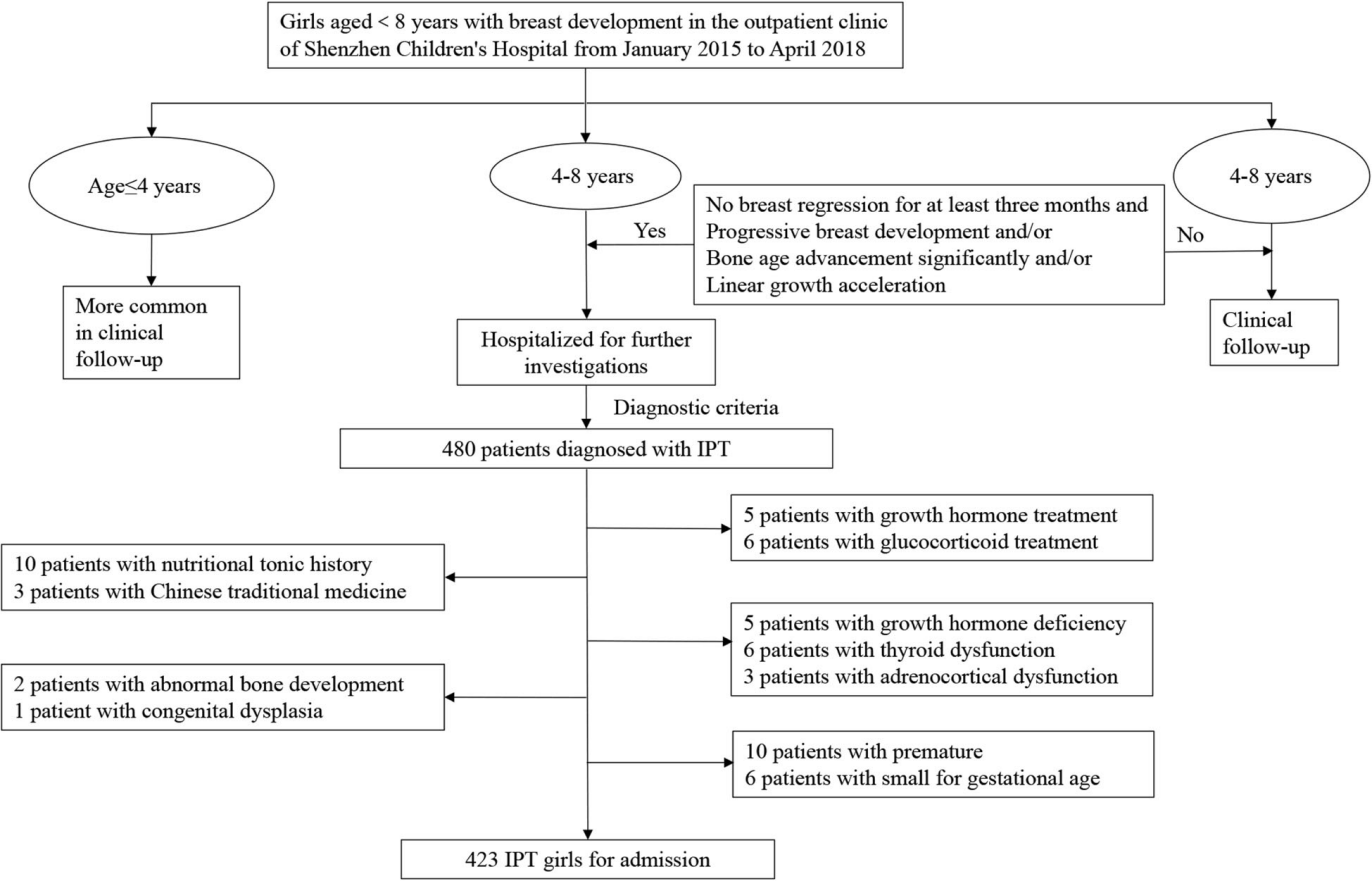
Flow chart of study population selection

Children with history of hormone treatment, history of intaking nutritional tonic and Chinese traditional medicine, other endocrine disease (including growth hormone (GH) deficiency, thyroid dysfunction, adrenocortical dysfunction or hyperactivity), abnormal bone development, congenital dysplasia, premature birth and small for gestational age were excluded. Ultimately, a total of 423 participants were enrolled in the study (Fig. 1).

According to the values of BA-CA, the participants were divided into the advanced BA group (BA-CA ≥ 2 years) and the control group (BA-CA < 2 years). According to body mass index (BMI) [8], the participants were classified into three groups: normal weight (BMI *P*_5_**~***P*_85_ or BMI standard deviation score (SDS) -1.65 to <1.04), overweight (BMI *P*_85_**~***P*_95_ or BMI SDS 1.04 to < 1.65) and obesity (BMI ≥ *P*_95_ or BMI SDS ≥ 1.65). IPT girls with normal weight were then subdivided into two groups separately according to the levels of serum insulin-like growth factor-1 (IGF-1) SDS and dehydroepiandrosterone sulfate (DHEAS) SDS.

Height and weight were measured, and BMI was calculated as body weight (kg)/height (m^2^). These values were expressed as age- and sex-specific SDS based on national survey data in 2005 [8, 9]. Breast development was staged according to the Tanner criteria. If the Tanner stages of bilateral breast development were different, the mature side was recorded. Hypothalamic-pituitary-gonadal axis function was evaluated by basal sexual hormone levels and the GnRH stimulation test (gonadorelin injections at 2.5-3.0 µg/kg, with no more than 100 µg administered at a time). Other hormones, including oestradiol, DHEAS, androsterone, sex hormone binding globulin (SHBG), IGF-1 and insulin, were evaluated by enzyme-enhanced chemiluminescence assays (Siemens IMMULITE 2000, Munich, Germany). IGF-1 and DHEAS levels were standardized according to reference data for different ages in China [10, 11]. Lipids, including triglycerides, total cholesterol, high-density lipoprotein and low-density lipoprotein, were measured using enzymatic end-point method.

We used automated artificial intelligence system (Yitu Healthcare, Hangzhou, China) to evaluate BA on a radiograph of the left hand. This product was version 3 of the Intelligent Diagnosis System for Child Growth and Development, which utilized RUS-CHN. All participants underwent a pelvic ultrasound examination (GE Logic E9 ultrasound machine with a 3.5–12 MHz convex probe and a 9–12 MHz linear probe).

### Statistical analysis

The t-test was used to compare between two groups for the following continuous measures: height (SDS), weight (SDS), BMI (SDS), and BA-CA. Data regarding age at onset, CA, disease duration, uterine length, ovarian volume, basic and peak LH, peak LH/peak FSH, DHEAS (SDS), IGF-1 (SDS), androsterone, insulin, SHBG, oestradiol and lipids were clearly skewed, so median and quartiles were used for these numerical variables. Those differences between two groups were compared using the Mann-Whitney U test. The association between significant BA advancement and breast Tanner stage as well as status of obesity or overweight were calculated using the chi-square test.

In order to identify independent risk factors affecting significantly advanced BA, we performed a binary logistic regression analysis (forward LR stepwise method) with the advanced BA of more than 2 years above CA (yes/no) as the dependent variable. The independent variables were those shown to be significant (*p* < 0.05) in the comparison between two groups (disease duration, BMI SDS, DHEAS SDS, IGF-1 SDS, androsterone, insulin, SHBG, uterine length), significant factors in previous studies (oestradiol), and other factors that may be clinically correlated (basic LH, peak LH, ratio of peak LH to peak FSH, ovarian volume).

A multiple linear regression model was then used to predict the factors most likely to advance BA, with BA-CA as the dependent variable. After testing the assumptions, we entered the factors shown to be significant (*p* < 0.05) in the comparison between two groups (BMI SDS, DHEAS SDS, IGF-1 SDS, insulin, SHBG, uterine length) and others that may be clinically correlated (peak LH, ratio of peak LH to peak FSH, ovarian volume) as the independent variables.

Statistical analyses were performed using SPSS version 23.0. We considered *p*-value < 0.05 as statistically significant for all analyses.

## Results

### General characteristics 

Among the 423 patients, the BA ranged from 4.60 to 12.50 years, and the BA-CA value, an index of BA advancement, ranged from − 0.87 to 4.18 years. Subjects were divided into the advanced BA group (BA-CA ≥ 2 years) and the control group (BA-CA < 2 years). There were 206 subjects (48.7%) in the advanced BA group (BA-CA, 2.63 ± 0.46 years) and 217 in the control group (BA-CA, 1.21 ± 0.59 years).

### Factors influencing BA advancement

There were no differences in the age of onset, CA at IPT diagnosis or Tanner stage between the two groups (Table 1). Compared to the control group, the advanced BA group had a higher BMI SDS (*p* < 0.001) and a lower height SDS for BA (*p* < 0.001). Serum DHEAS SDS, IGF-1 SDS, androstenedione and fasting insulin were significantly higher in the advanced BA group than those in the control group (all *p* < 0.001). However, SHBG was lower in the advanced BA group (*p* < 0.001). The uterine diameter was longer in the advanced BA group (*p* = 0.009). The biochemical and imaging characteristics of the study groups are described in Table 2.

**Table 1:** Physical characteristics of the study population

	Advanced BA group	Control group	*p*-value
Number of girls	206 (48.7%)	217 (51.3%)	-
Chronological age (years)	7.56 (6.90, 7.95)	7.72 (7.06, 8.04)	0.189
Disease duration (years)	0.50 (0.25, 1.00)	0.33 (0.25, 0.75)	0.029
Age at onset (years)	6.86 (6.12, 7.41)	7.13 (6.37, 7.60)	0.082
Height-for-age (cm)	130.77 ± 6.28	126.76 ± 7.14	< 0.001
Height-for-age SDS	1.16 ± 0.86	0.36 ± 0.88	< 0.001
Height-for-BA SDS	-1.45 ± 0.72	-0.79 ± 0.95	< 0.001
Genetic target height SDS	-0.48 ± 0.80	-0.64 ± 1.39	0.165
HtBA SDS - THt SDS	-0.97 ± 0.80	-0.16 ± 1.42	< 0.001
Weight-for-age (kg)	30.06 ± 5.90	26.58 ± 4.93	< 0.001
Weight-for-age SDS	1.46 ± 1.05	0.64 ± 0.96	< 0.001
BMI	17.45 ± 2.39	16.44 ± 2.06	< 0.001
BMI SDS	1.16 ± 1.10	0.64 ± 1.07	< 0.001
Breast Tanner stage			0.083
2	137 (32.4%)	161 (38.1%)	
3	69 (16.3%)	56 (13.2%)	
Birth weight (kg)	3.25 ± 0.40	3.24 ± 0.42	0.762

Categorical variables are presented as number (%, percentage). Continuous variables with normal distribution are presented as the mean ± standard deviation, and variables with non-normal distribution are presented as the median and quartiles

*BA* Bone age, *SDS* Standard deviation score, *HtBA SDS* THt SDS height-for-BA SDS minus genetic target height SDS, *BMI* Body mass index

**Table 2:** Clinical characteristics of the study population

	Advanced BA group	Control group	*p*-value
Basic LH (IU/L)	0.24 (0.15, 0.42)	0.24 (0.13, 0.44)	0.902
Peak LH (IU/L)	3.49 (2.52, 4.18)	3.57 (2.80, 4.16)	0.301
Peak LH/peak FSH	0.22 (0.17, 0.29)	0.21 (0.16, 0.29)	0.316
DHEAS (µg/dL)	42.85 (23.78, 60.83)	29.50 (16.80, 52.40)	< 0.001
DHEAS SDS	1.94 (0.46, 4.15)	0.85 (-0.14, 2.47)	< 0.001
Androsterone (ng/mL)	0.30 (0.15, 0.48)	0.15 (0.15, 0.35)	< 0.001
Insulin (µIU/mL)	6.68 (5.08, 9.37)	5.50 (3.56, 8.10)	< 0.001
SHBG (nmol/L)	58.15 (41.75, 75.23)	71.70 (52.80, 89.90)	< 0.001
IGF-1 (ng/mL)	238.00 (190.00, 300.25)	204.00 (168.00, 241.00)	< 0.001
IGF-1 SDS	0.44 (-0.30, 1.29)	-0.27 (-0.73, 0.48)	< 0.001
Oestradiol (pmol/L)	10.00 (10.00, 20.00)	10.00 (10.00, 10.00)	0.257
Triglyceride (mmol/L)	0.80 (0.61, 1.04)	0.85 (0.63, 1.05)	0.366
Total cholesterol (mmol/L)	3.76 (3.37, 4.35)	3.86 (3.49, 4.34)	0.299
HDL (mmol/L)	1.23 (1.06, 1.38)	1.23 (1.05, 1.42)	0.646
LDL (mmol/L)	2.27 (1.96, 2.63)	2.36 (2.07, 2.69)	0.215
Uterine length (cm)	3.41(3.08,3.67)	3.27(2.93,3.49)	0.009
Ovarian volume (ml)	1.23(0.82,1.74)	1.13(0.78,1.76)	0.389

Data presented as the median and quartiles

*BA* Bone age, *LH* Luteinizing hormone, *FSH* Follicle-stimulating hormone, *DHEAS* Dehydroepiandrosterone sulfate, *SDS* Standard deviation score, *SHBG* Sex hormone binding globulin, *IGF-1* Insulin-like growth factor-1, *HDL* High-density lipoprotein, *LDL* Low-density lipoprotein

### Independent risk factors affecting significantly advanced BA 

The results of binary logistic regression analysis (forward LR stepwise method) were presented in Table 3. Serum IGF-1 SDS (OR = 1.926, *p*<0.001), BMI SDS (OR = 1.427, *p* = 0.001) and DHEAS SDS (OR = 1.131, *p* = 0.005) were independent risk factors for significantly advanced BA. For each 1 SD increase in IGF-1 SDS, BMI SDS and DHEAS SDS above normal, the risk of significantly advanced BA increased by 92.6%, 42.7% and 13.1%, respectively.

**Table 3:** Independent risk factors affecting significantly advanced BA

	OR	95%CI	*p*-value
IGF-1 SDS	1.926	1.524–2.436	<0.001
DHEAS SDS	1.131	1.039–1.232	0.005
BMI SDS	1.427	1.160–1.755	0.001

*OR* Odds ratio, *CI* Confidence interval, *SDS* Standard deviation score, *IGF-1* Insulin-like growth factor-1, *DHEAS* Dehydroepiandrosterone sulfate, *BMI* Body mass index

### A model for predicting BA-CA 

A multiple linear regression model was generated to predict BA-CA. The regression model was statistically significant (Table 4): F = 10.425, *p* < 0.001. The model could be expressed as presented in equation: Y(BA-CA) = 1.708 + 0.168× (IGF-1 SDS) + 0.131× (BMI SDS) + 0.049× (DHEAS SDS). Serum IGF-1 SDS was the top predictor of BA-CA, followed by BMI SDS and DHEAS SDS. This model accounted for 19.3% of the variance in BA-CA.

**Table 4:** A model for predicting BA-CA

	Beta	Standard error	VIF	t	*p*-value	R2
(Constant)	1.708	0.386		4.431	< 0.001	
IGF-1 SDS	0.168	0.041	1.171	4.119	< 0.001	
DHEAS SDS	0.049	0.015	1.141	3.241	0.001	
BMI SDS	0.131	0.045	1.521	2.924	0.004	
Model	Y(BA-CA) = 1.708 + 0.168× (IGF-1 SDS) + 0.131× (BMI SDS) + 0.049× (DHEAS SDS)				< 0.001	19.3

*IGF-1* Insulin-like growth factor-1, *SDS* Standard deviation score, *BMI* Body mass index, *DHEAS* Dehydroepiandrosterone sulfate

### Comparisons of advanced BA in the subgroup

According to BMI, 423 patients were classified into three groups: normal weight (56.03%, n = 237), overweight (19.15%, n = 81) and obesity (24.82%, n = 105). The proportions of BA advancement in the three groups were 42.6%, 43.2% and 66.7%, respectively. The difference was statistically significant (*χ*^2^ = 18.055, *p* < 0.001). It indicated that IPT girls in the obese group had a higher risk of significantly advanced BA. While excluding the influence of obesity/overweight, a high proportion of IPT girls with normal weight still had significantly advanced BA.

In IPT girls with normal weight, we compared the differences of IPT girls with higher serum IGF-1 (IGF-1 SDS ≥ 0 SD) and lower IGF-1 (IGF-1 SDS < 0 SD), and compared those with higher serum DHEAS for age (DHEAS SDS ≥ 2 SD) to those with normal (DHEAS SDS < 2 SD). As is shown in Tables 5 and 6, there was no significant difference in BMI SDS between the two groups. After excluding interference by obesity, higher IGF-1 SDS (*p* = 0.009) and DHEAS SDS (*p* = 0.003) independently affect BA advancement.

**Table 5:** Comparison of IGF-1 SDS in normal weight subgroup

	IGF-1 SDS<0SD	IGF-1 SDS ≥ 0SD	*p*-value
Number of girls	120	110	-
BA-CA (years)	1.60 ± 0.83	1.90 ± 0.93	0.009
BMI SDS	0.05 ± 0.68	0.17 ± 0.66	0.177

Data presented as mean ± standard deviation

*IGF-1* Insulin-like growth factor-1, *SDS* Standard deviation score, *BA-CA* Bone age minus chronological age, *BMI* Body mass index

**Table 6:** Comparison of DHEAS SDS in normal weight subgroup

	DHEAS SDS<2SD	DHEAS SDS ≥ 2SD	*p*-value
Number of girls	161	76	-
BA-CA (years)	1.64 ± 0.86	2.00 ± 0.87	0.003
BMI SDS	0.10 ± 0.69	0.13 ± 0.66	0.759

Data presented as mean ± standard deviation

*DHEAS* Dehydroepiandrosterone sulfate, *SDS* Standard deviation score, *BA-CA* Bone age minus chronological age, *BMI* Body mass index

## Discussion

In this study, nearly half of the IPT girls confirmed by GnRH stimulation test aged 4–8 years had significantly advanced BA, resulting in impaired height for BA. The high proportion of IPT girls with advanced BA in our study may be related with the criteria for requiring further investigations. In addition, one inclusion criterion was age at onset of 4–8 years, which excluded interference by mini-puberty and may increase the probability of observing advanced BA. In contrast to our study, previous studies on IPT have included patients in two categories: patients aged 1–4 years with IPT or all patients younger than 8 years with IPT.

IPT is typically seen in girls with normal growth and normal bone maturation [12]. Oppositely, BA of patients with central precocious puberty (CPP) is generally more advanced than their CA. However, the degree of BA advancement cannot be synchronized with the process of puberty [13]. Patients with CPP accompanied by short disease course or slow pubertal process may not have obvious BA advancement. Thus, the IPT girls with BA advancement should be identified and deserved of more attention.

Advanced BA is found to be influenced by a number of factors in many researches, such as oestrogens [13], androgens [14], GH [15], thyroid hormone [16], nutrition [17], genetics [18] and so on. These studies, however, were mainly aimed at children with obesity. Our study focused on IPT girls and showed that IGF-1, DHEAS and BMI were the most important factors affecting BA advancement, especially the first two independent of BMI.

The important finding of the present study is that higher serum IGF-1 SDS independently affects BA advancement in girls with IPT. For each 1 SD increase in IGF-1, the risk of significantly advanced BA increases by 92.6%. The GH/IGF-1 axis is critically important for regulating bone maturation. Increased GH secretion augments circulating IGF-1 levels, inducing the proliferation and differentiation of chondrocytes in the epiphyseal growth plate. Reinehr et al. [19] studied 356 children with obesity and postulated that IGF-1 plays a role in advanced skeletal maturation, independent of sex or pubertal stage. Oppositely, other studies showed no such association [14, 20].

In addition, excluding the interference of obesity, IGF-1 might be elevated before initiation of puberty. A longitudinal study of 526 healthy children aged 6–16 years showed that the elevated level of circulating IGF-1 occurred 2 years earlier than that of LH level [21]. It may be explained by the expression of IGF-1 receptors in GnRH neurons. Yan et al. [22] infused IGF-1 and antagonist of IGF-1 receptors into the third ventricle of ovariectomized female rats. They found that IGF-1 receptors are required for estradiol activation of GnRH neurons and for robust GnRH release. Thus, elevated serum IGF-1 levels in IPT girls may have a predictive effect on BA advancement and initiation of puberty.

Similar to the findings by Kwon et al. [23] and others [20, 24], our study showed a positive association between serum DHEAS SDS and advanced BA. This relationship is likely due to the higher adrenal androgen levels in children with obesity; these increased levels are believed to be responsible for the advanced BA. Giving that obesity is also an important factor in BA advancement, we re-analyzed serum DHEAS SDS and advanced BA in IPT girls with normal weight and made the regression analyses. The present study showed that the levels of serum DHEAS are positively associated with BA advancement independent of BMI, which indicates that attention should be paid to the levels of DHEAS even among non-obese prepubertal children.

On the other hand, DHEAS is thought to be a marker of adrenal androgen secretion and adrenarche. It is suggested that whether BA advancement of IPT girls with high level of DHEAS is associated with relatively active adrenal function. However, there are no unified diagnostic criteria for adrenarche at present. The clinical features of adrenarche are considered as development of axillary hair, acne, oily skin and hair, and adult body odor, but patients with these distinguishing features were excluded from the present study population. Su et al. [10] reported that among individuals older than 6 years, serum DHEAS levels increased along with pubertal development in individuals of the same sex in the same age group. It was demonstrated that adrenarche and gonadarche might possibly be related to each other. The higher level of DHEAS may be presented with IPT girls. Therefore, DHEAS could theoretically have independent role at bone maturation of IPT children.

As expected, in this cohort of IPT girls, obesity was highly associated with advanced BA. This is in line with previous studies. Klein et al. [25] studied 167 children across all pubertal stages and from normal weight to obesity and reported that almost 25% of the obese children had BA advancement by more than 2 SD for CA and that 33% had BA advancement by more than 2 years above CA and up to 6.5 years. Another cross-sectional study of 66 prepubertal children with premature adrenarche and control subjects reached similar conclusions [20]. The above studies included children at different stages of puberty, which may be influenced by the sexual maturation. Our study cohort only included children with IPT, a relatively homogeneous population, to analyze the relationship between obesity and advanced BA again. In addition, obesity is considered one possible cause of IPT [26]. The effect of BMI on thelarche is likely related to fat mass and the resulting increase in leptin secretion, which is known to affect hypothalamic GnRH-secreting neurons [27]. Thus, more children with IPT may be obese, and the risk of advanced BA may be higher in IPT children.

As shown in multiple linear regression model, IGF-1 SDS, BMI SDS and DHEAS SDS could explain 19.3% of the variance in advanced BA, suggesting that more unknown factors not included in this study might contribute to advanced BA. Similarly, in a study of 66 prepubertal children with obesity and premature adrenarche, an exploratory stepwise regression model showed that weight, oestradiol and DHEAS were the strongest predictors of BA/CA, accounting for 24% of the variance [20]. Another study in 101 obese children generated a backward regression analysis model in which sex, DHEAS SDS, and age explained 27% of the total variance in BA SDS [24]. Therefore, there may be other underlying causes of advanced BA, including genetic causes such as heterozygous mutations in ACAN [28] and extrinsic factors such as exposure to endocrine-disrupting chemicals [29]. Future studies should clarify the roles of these factors in bone maturation.

Some limitations of our study need to be considered. First, blood samples were not collected in the same batch in this retrospective study. Second, there was no regular follow-up of this group of children. A long-term longitudinal design would offer the opportunity to monitor breast development and BA and determine the final adult height. Therefore, further studies are still needed.

## Conclusions

Girls with IPT confirmed by GnRH stimulation test aged 4–8 years might have advanced BA of more than 2 years above CA, resulting in impaired height for BA. Obesity was highly associated with advanced BA. Age-specific serum IGF-1 SDS and DHEAS SDS were risk factors for BA advancement independent of BMI. For each 1 SD increase, the risk increased by 92.6% and 13.1%, respectively.

## Data Availability

The datasets used and analysed during the present study are available from the corresponding author on reasonable request.

## Abbreviations

IPT: Isolated premature thelarche
BA: Bone age
CA: Chronological age
BA-CA: Bone age minus chronological age
BMI: Body mass index
SDS: Standard deviation score
LH: Luteinizing hormone
FSH: Follicle-stimulating hormone
GnRH: Gonadotropin-releasing hormone
GH: Growth hormone
DHEAS: Dehydroepiandrosterone sulfate
SHBG: Sex hormone binding globulin
IGF-1: Insulin-like growth factor-1
CPP: Central precocious puberty
